# HM-GAT: Vehicle Trajectory Prediction Based on Hierarchical Mamba and Graph Attention Networks

**DOI:** 10.3390/s26196332

**Published:** 2026-10-07

**Authors:** Qiong Shi, Xinjie Zhang, Hong Zhao, Qingde Li

**Affiliations:** 1 School of Computer Science and Technology, North University of China, Taiyuan 030051, China; sz202407045@st.nuc.edu.cn (X.Z.);; 2 School of Computer Science, University of Hull, Hull HU 67RX, UK

**Keywords:** vehicle trajectory prediction (VTP), Mamba network, vehicle interaction, graph attention network (GAT), spatio-temporal fusion

## Abstract

In highly dynamic and interactive vehicular environments, accurate and efficient trajectory prediction is critical for driving safety and traffic efficiency. To overcome the limitations of existing methods, including degradation of long-range historical context, high computational cost for long sequences, attention dispersion in spatial interactions, and limited explicit modeling of future interactions, this paper proposes a Hierarchical Mamba Graph Attention Network (HM-GAT) for vehicle trajectory prediction. A Mamba-based selective state-space model (SSM) encoder with linear complexity is employed to capture nonlinear temporal dynamics and transient maneuvering behaviors. For spatial modeling, a heterogeneous topology-driven dual-layer graph attention mechanism, combined with adaptive sparse filtering and a foresighted future graph, enables precise identification of neighboring vehicles with potential collision risks. The proposed architecture integrates global intention exploration, topology bridging, and residual refinement in a coarse-to-fine manner, achieving effective spatio-temporal decoupling. Experimental results show that HM-GAT reduces the average RMSE by 33.1% relative to the best-performing baseline on NGSIM, achieves competitive prediction accuracy on Argoverse 2 without HD-map priors, and achieves lower inference latency than the compared baseline methods on both datasets.

## 1. Introduction

Vehicle Trajectory Prediction (VTP) for road traffic and autonomous driving is a critical bridge between environmental perception and autonomous decision-making in the Internet of Vehicles (IoV) [1,2,3]. Its primary objective is to infer the future trajectory of a target vehicle over a given prediction horizon based on historical observations of the target vehicle and its surrounding traffic participants, together with local road topology information. In real-world traffic scenarios, a driver’s decision-making process is influenced not only by intrinsic driving intentions, such as aggressive lane changing, conservative car-following, and emergency obstacle avoidance, but also by external factors, including interactions with neighboring vehicles and traffic signal states [4]. The nonlinear coupling of these internal and external influences results in highly multimodal trajectory distributions and significant predictive uncertainty within complex spatial topologies [5].

Conventional kinematic approaches, including constant-velocity/acceleration models and standard Kalman-filter-based formulations, primarily rely on predefined motion assumptions and generally provide limited explicit representation of complex multi-agent interactions. More advanced filtering variants can incorporate interaction-related information and remain important interpretable baselines [6]. Subsequently, conventional machine learning methods, including Support Vector Machines (SVMs) and Hidden Markov Models (HMMs) [7], improved trajectory modeling by incorporating statistical features. However, their performance remains heavily dependent on handcrafted feature engineering, making it difficult to fully capture the complex nonlinear patterns embedded in high-dimensional trajectory data. In recent years, the rapid advancement of deep learning has substantially reduced the dependence on manually designed features through data-driven representation learning. Owing to its powerful representation-learning capability, deep learning has become the dominant paradigm for vehicle trajectory prediction.

Although deep learning-based trajectory prediction models have achieved remarkable progress on public benchmark datasets, they still face several critical limitations when deployed in complex real-world traffic environments.

First, long-range temporal modeling remains challenging in terms of both historical-context preservation and computational efficiency. Recurrent neural networks (RNNs), which are widely adopted for sequential modeling, process temporal observations sequentially and compress historical information into recurrent hidden states. Related applications in transportation also employ recurrent architectures for temporal feature extraction; for example, Wang et al. [8] combined CNN and LSTM components for rail-corrugation diagnosis using onboard vibration measurements. When processing long historical observation sequences, they may suffer from gradient attenuation and degradation of long-range historical context, limiting their ability to capture drivers’ transient maneuvering intentions [9]. Transformer-based architectures have recently improved long-range dependency modeling by introducing self-attention mechanisms with a global receptive field [10,11,12]. However, for a temporal sequence of length T, full self-attention requires $O(T^{2})$ pairwise attention computation, which can incur substantial computational overhead for long observation sequences and multi-agent prediction settings, potentially increasing inference latency.

Second, spatial interaction modeling is challenged by densely distributed interaction information and the limited explicit modeling of future interaction relationships. In dense traffic scenarios, the Softmax operator employed in conventional Graph Attention Networks (GATs) assigns normalized nonzero attention weights to all retained neighboring nodes [13,14]. When the interaction graph contains numerous weakly relevant neighbors, the absence of explicit structural sparsification may reduce the concentration of attention on a small subset of critical interactions. Moreover, many existing methods construct interaction graphs primarily from observed or current vehicle configurations [15]. Although such graphs can encode historical interactions and support future inference, their edge structures do not explicitly represent interaction relationships that may emerge later within the prediction horizon. This current-to-future topology mismatch can limit the modeling of potential future conflicts and affect long-horizon prediction accuracy.

Third, long-horizon prediction is affected by multimodal uncertainty and, for autoregressive decoders, accumulated prediction errors. When a model is forced to generate a single deterministic trajectory, the optimization objective tends to average multiple plausible future intentions, often resulting in unrealistic trajectories that lie between feasible driving paths and even across lane boundaries, a phenomenon more appropriately characterized as mode averaging or regression toward the conditional mean [16]. This limitation is distinct from error accumulation in autoregressive prediction. For models employing autoregressive decoding, small one-step prediction errors may alter the subsequent input distribution and accumulate over successive prediction steps, eventually leading to substantial deviations from the ground-truth trajectory at the end of the prediction horizon. Multimodal prediction alleviates the deterministic averaging problem by representing multiple plausible future hypotheses, but accurate mode selection and stable long-horizon refinement remain challenging.

To address the aforementioned limitations, this paper departs from the conventional paradigm of single-stage sequential decoding and current-topology-only interaction modeling and proposes a novel coarse-to-fine hierarchical trajectory prediction framework, namely the Hierarchical Mamba and Graph Attention Network (HM-GAT). The proposed model integrates a linear-complexity Mamba network with an enhanced graph attention network for spatio-temporal fusion and further constructs cascaded layers for hierarchical coarse-to-fine optimization.

The main contributions of this paper are summarized as follows:

- A Mamba-based temporal dynamics reconstruction method is introduced for vehicle motion encoding, which employs selective state-space modeling to capture nonlinear temporal dynamics and transient vehicular maneuvers with computational complexity that scales linearly with temporal sequence length. Compared with full temporal self-attention, this design provides a more computationally efficient mechanism for modeling long historical sequences.
- An adaptive sparse interaction-enhanced graph attention mechanism driven by heterogeneous topology is proposed. A dual-layer spatial receptive field is designed to separate interaction aggregation based on the observed current topology from refinement guided by an anticipatory topology constructed from predicted future trajectories. Furthermore, a sparsification operator is introduced to reduce weakly relevant interaction edges before attention normalization, with the aim of concentrating graph aggregation on more informative neighboring interactions. The combination of current and anticipatory topologies is designed to alleviate the mismatch between observed interaction structures and potential future interactions.
- A coarse-to-fine hierarchical spatio-temporal optimization architecture is proposed. The framework incorporates a cascaded network composed of a global intention exploration layer, an anticipatory topology bridging layer, and a microscopic residual refinement layer. By jointly optimizing multiple objectives through a unified multi-task loss function, the proposed architecture progressively refines coarse multimodal trajectory hypotheses and is designed to mitigate long-horizon prediction error growth, thereby improving prediction stability and accuracy.
- Comprehensive experiments are conducted on the publicly available NGSIM and Argoverse 2 datasets to evaluate the effectiveness of the proposed framework. Experimental results demonstrate that HM-GAT achieves strong long-horizon prediction performance on NGSIM and competitive accuracy on AV2 without HD-map priors, while maintaining lower inference latency than the compared baseline methods on both datasets.

## 2. Related Work

The core of current vehicle trajectory prediction research lies in the joint modeling of spatio-temporal dependencies. Existing methods can be broadly categorized into two perspectives: temporal dependency encoding and spatial interaction modeling.

### 2.1. Temporal Dependency Encoding

Temporal dependency encoding aims to capture the dynamic evolution patterns of non-stationary sequential trajectories and map raw physical motion into high-dimensional latent representations. To this end, recurrent neural networks (RNNs) and their gated variants, including Long Short-Term Memory (LSTM) and Gated Recurrent Unit (GRU), have been widely adopted due to their inherent capability in modeling variable-length sequences, establishing the foundation of temporal feature extraction [17,18,19,20]. Building upon these models, classical architectures such as CS-LSTM [21] and TCN-LSTM [18] further demonstrate strong capability in capturing continuous trajectory dependencies.

However, the inherently sequential computation of recurrent architectures limits temporal parallelism, while long historical sequences can increase optimization difficulty because information must be propagated and compressed through recurrent hidden states. Consequently, recurrent models may suffer from gradient attenuation and degradation of long-range historical context when modeling extended temporal dependencies. To improve long-range dependency modeling and temporal parallelism, Transformer-based architectures leveraging self-attention mechanisms have been introduced into trajectory prediction tasks [22,23,24]. Transformers provide direct pairwise access between temporal tokens through self-attention, facilitating the modeling of long-range dependencies and contributing to improved trajectory prediction performance. Nevertheless, their quadratic time and space complexity with respect to sequence length makes them difficult to deploy in edge-based vehicular systems with strict real-time constraints.

To efficiently capture long-range temporal dependencies, selective state-space models represented by Mamba were proposed by Gu and Dao in 2023 [25], providing an efficient alternative to architectures relying primarily on quadratic self-attention. Mamba introduces a data-dependent parameterization mechanism, enabling adaptive modulation of the discretization step and state-space parameters according to the input sequence, thereby supporting selective temporal information propagation. Recently, Huang et al. [26] proposed Trajectory Mamba, an efficient autonomous-driving trajectory forecasting framework based on selective state-space modeling. The model combines joint polyline encoding with cross-state-space attention to capture static and dynamic scene context while reducing the computational burden associated with attention-heavy architectures. Li et al. [27] subsequently proposed the Future-Aware Interaction Network (FINet), which introduces potential future trajectories into the scene-encoding stage and employs Mamba for both spatial interaction modeling and temporal trajectory refinement. To adapt Mamba to unordered spatial elements, FINet further introduces an adaptive reordering strategy that transforms spatial tokens into a structured sequence. Liu et al. [28] proposed GAMDTP, which integrates self-attention and Mamba-SSM through a gated mechanism within graph-based interaction layers and adopts a two-stage proposal-and-refinement strategy with trajectory scoring for dynamic motion prediction.

Despite the effectiveness of these recent Mamba-based motion forecasting methods, HM-GAT differs from them in temporal modeling granularity, spatial interaction design, and hierarchical optimization logic. Trajectory Mamba [26] mainly employs selective state-space modeling and cross-state-space attention within an efficient encoder–decoder framework, whereas FINet [27] extends Mamba to spatial interaction modeling through adaptive token reordering and incorporates potential future trajectories into scene encoding. GAMDTP [28] combines self-attention and Mamba-SSM within graph-based interaction layers and adopts a proposal–refinement prediction strategy. In contrast, HM-GAT explicitly separates temporal and spatial reasoning into a hierarchical coarse-to-fine framework. At the temporal level, Mamba encoders are employed in both the coarse intention exploration stage and the subsequent trajectory refinement stage. At the spatial level, HM-GAT employs graph attention over two heterogeneous interaction domains: an observed current topology for coarse interaction modeling and an anticipatory topology constructed from predicted future trajectories for subsequent refinement. In addition, topology-conditioned sparse filtering is introduced before graph attention to suppress weakly relevant interaction edges. At the optimization level, multimodal candidate generation, future-topology construction, and residual refinement are sequentially organized into the FLN–ATL–SLN pipeline.

### 2.2. Spatial Interaction Modeling

In real-world traffic scenarios, interactions among vehicles inherently form highly dynamic non-Euclidean topological structures. Recent studies have demonstrated that Graph Neural Networks (GNNs) provide a flexible mechanism for representing non-Euclidean interaction relationships among traffic participants and have therefore become a widely used class of methods for vehicle interaction modeling. For instance, GRIP++, proposed by X. Li et al. [29], constructs a hybrid topology by integrating static and dynamic graphs to enhance vehicle interaction modeling. GSTCN, proposed by Z. Sheng et al. [30], builds a weighted adjacency matrix based on inverse inter-vehicle distances and employs Graph Convolutional Networks (GCNs) for spatial interaction feature aggregation. However, when coupled with fixed or manually constructed graph structures, conventional GCN-based formulations may have limited flexibility in adapting interaction strengths to rapidly evolving relationships among traffic participants. To improve dynamic interaction modeling, Graph Attention Networks (GATs) have been introduced to enable adaptive weight allocation. For example, STA-LSTM, proposed by L. Lin et al. [31], decouples spatial and temporal attention mechanisms to quantify the influence of neighboring vehicles. P. Cong et al. [32] incorporated a collision risk-based dynamic drivable region assessment strategy into DACR-AMTP, while the FHIF model proposed by Z. Zuo et al. [33] leverages multimodal bilinear pooling to fuse historical trajectory information with interaction-field features. To address the potential dilution of interaction attention in densely connected scenarios, P. Qian et al. [34] designed a learnable sparse interaction module in the SSTT framework, enabling the model to focus on critical targets by filtering redundant interaction edges.

Although these methods have achieved promising results in topological interaction modeling, many existing approaches primarily construct interaction structures from observed or current vehicle configurations. Although such graph representations can encode historical interactions and support future-motion inference, their edge structures do not explicitly represent interaction relationships that may emerge later within the prediction horizon. Consequently, a mismatch may arise between the interaction topology encoded from current observations and the interaction relationships that develop during future motion. Moreover, in densely connected graphs, numerous weakly relevant neighbors may reduce the concentration of attention on a small number of highly informative interactions, thereby limiting prediction performance.

It should be noted that sparse interaction modeling itself has already been explored in previous studies such as SSTT [34]. Therefore, the distinction of HM-GAT does not lie in sparsification alone. Instead, the proposed framework integrates adaptive sparse interaction modeling with two heterogeneous graph domains: the observed current topology used for coarse intention exploration and an anticipatory topology constructed from predicted future trajectories for subsequent refinement. By combining topology-conditioned interaction filtering with hierarchical temporal encoding and coarse-to-fine residual refinement, HM-GAT is designed to model both currently observed interactions and interaction relationships that may emerge within the future prediction horizon.

## 3. HM-GAT Model

### 3.1. Problem Formulation

Suppose that in the current traffic scenario within the historical observation time window $T_{h}$, the model performs neighbor selection based on the local spatial perception range of the target vehicle. Specifically, a rectangular interaction region centered on the target vehicle is defined with a longitudinal range of ±20 m and a lateral range of ±5 m. These bounds are fixed engineering settings informed by the highway and urban road scenarios represented in the datasets, together with the lane structure and typical car-following and lane-changing interactions encountered in real traffic. The selected ranges prioritize local vehicle interactions while keeping the candidate interaction graph compact. A surrounding vehicle j is regarded as a potential interacting neighbor of target vehicle i when $|\Delta x_{ij}^{t}|\leq 20,\;|\Delta y_{ij}^{t}|\leq 5$ where $\Delta x_{ij}^{t}=x_{j}^{t}-x_{i}^{t},\;\Delta y_{ij}^{t}=y_{j}^{t}-y_{i}^{t}$, denote the relative longitudinal and lateral positions respectively. For an arbitrary target vehicle i, its planar position at time step t is represented as $p_{i}^{t}=(x_{i}^{t},y_{i}^{t})$. The first-order velocity $v_{i}^{t}$, second-order acceleration $a_{i}^{t}$, and headway distance $d_{i,head}^{t}$ are further incorporated to describe its motion state. Consequently, the input state vector of vehicle ii at time t is represented as $x_{i}^{t}={\left[\begin{array}{ccccc} x_{i}^{t}, & y_{i}^{t}, & v_{i}^{t}, & a_{i}^{t}, & d_{i,head}^{t} \end{array}\right]}^{⊤}$. Here $v_{i}^{t}$ and $a_{i}^{t}$ are scalar velocity and acceleration values, respectively, while lateral-motion characteristics are represented by the temporal variation of $y_{i}^{t}$. The headway feature $d_{i,head}^{t}$ is obtained from the Space_Headway attribute of the NGSIM dataset and describes the spacing between vehicle i and its preceding vehicle in the same lane. For samples in which no valid preceding vehicle exists within the modeled interaction region, the headway feature is assigned a value of 20 m. The complete historical state sequence of vehicle ii is represented as $X_{i}=\left[\begin{array}{c} x_{i}^{t-T_{h}+1},\ldots ,x_{i}^{t} \end{array}\right]\in R^{T_{h}\times 5}$. At the final observed time step, the spatial interaction topology is represented by a binary adjacency matrix $A^{curr}\in \{0,1{\}}^{N\times N}$:

(1) $$A_{ij}^{curr}=\{\begin{array}{c} 1,|\Delta x_{ij}^{t}|\leq 20\;and\;|\Delta y_{ij}^{t}|\leq 5,\\ 0,otherwise. \end{array}$$

Because the interaction condition is based on symmetric relative-distance constraints, $A_{ij}^{curr}=A_{ji}^{curr}$, and the resulting current interaction graph is therefore binary and undirected. Self-loops are introduced such that $A_{ii}^{curr}$ = 1, allowing each node’s own representation to participate in neighborhood aggregation. We formulate the task as a conditional multimodal trajectory-prediction problem. Given the historical state set X and the observed interaction topology, the objective is to predict M candidate trajectories for target vehicle i over the next $T_{f}$ time steps:

(2) $$p(Y_{i}|X,A^{curr})=\sum_{m=1}^{M}p_{i}^{\left(m\right)}\;\delta \left(Y_{i}-{\hat{Y}}_{i}^{\left(m\right)}\right)$$

where δ(⋅) denotes the Dirac delta function. The m-th candidate trajectory is ${\hat{Y}}_{i}^{\left(m\right)}=\left[\begin{array}{c} {\hat{y}}_{i,1}^{\left(m\right)},\ldots ,{\hat{y}}_{i,T_{f}}^{\left(m\right)} \end{array}\right]\in R^{T_{f}\times 2}$, and the corresponding probabilities satisfy $p_{i}^{\left(m\right)}\geq 0\;,\sum_{m=1}^{M}p_{i}^{\left(m\right)}=$ 1. Each candidate represents a learned latent future-motion hypothesis. The M modes are learned from trajectory data rather than manually predefined as fixed maneuver classes such as lane keeping or lane changing.

### 3.2. HM-GAT Model

A coarse-to-fine hierarchical prediction paradigm, namely the Hierarchical Mamba and Graph Attention Network (HM-GAT), is proposed in this study. The overall architecture is logically decomposed into three core cascaded modules: the First Layer Network (FLN), the Anticipatory Topology Layer (ATL), and the Second Layer Network (SLN). The FLN first generates multiple coarse future hypotheses, the ATL transforms model-predicted future motion into an anticipatory interaction topology, and the SLN subsequently refines the highest-probability coarse prediction. During training, ground-truth future trajectories are used only for loss supervision and are never used to construct the anticipatory graph.

1. FLN: Global Intent Exploration Layer. First, the input historical trajectories are projected into a high-dimensional representation through a linear embedding layer. A selective Mamba encoder with data-dependent dynamics is then employed to extract temporal dynamical features. Subsequently, the encoded features are fed into a dynamic graph attention layer, which performs spatial aggregation based on the current physical adjacency matrix $A_{\mathrm{curr}}$, thereby aggregating interaction information from currently relevant neighboring vehicles. Finally, learnable multimodal intention embeddings are incorporated to generate M coarse candidate trajectories ${\left\{{\hat{Y}}_{i}^{c,(m)}\right\}}_{m=1}^{M}$ and the corresponding mode probabilities ${\left\{p_{i}^{\left(m\right)}\right\}}_{m=1}^{M}$.
2. ATL: Anticipatory Topology Layer. To address the mismatch between the interaction topology observed at the current time and the interaction relationships that may emerge during the future prediction horizon, a bridging mechanism is introduced between the two prediction stages. For each vehicle i, the ATL selects the highest-probability Top-1 mode generated by the FLN: $m_{i}^{b}=\underset{m\in \{1,\ldots ,M\}}{\mathrm{arg max}}\;p_{i}^{\left(m\right)}$, and the corresponding coarse trajectory is ${\hat{Y}}_{i}^{b}={\hat{Y}}_{i}^{c,(m_{i}^{b})}$. The future trajectory of each neighboring vehicle is selected independently using the same Top-1 rule. Importantly, the same selection mechanism is applied during both training and inference. The ground-truth-matched mode $m_{i}^{*}$, introduced later for loss supervision, is not propagated to the ATL. Therefore, ground-truth future coordinates are never used to construct the anticipatory graph. Top-1 selection keeps the anticipatory topology aligned with the coarse prediction subsequently refined by the SLN. However, the remaining lower-probability hypotheses are not included in topology construction, and safety-relevant interactions arising exclusively under these alternative futures may therefore not be represented. For target vehicle i and neighboring vehicle j, the minimum scaled convergence distance over the prediction horizon is
  (3) $$D_{ij}^{fut}=\underset{\tau =1,\ldots ,T_{f}}{\min}{\left\|W_{scale}\left({\hat{y}}_{i,\tau }^{b}-{\hat{y}}_{j,\tau }^{b}\right)\right\|}_{2}$$
  where $W_{scale}=diag(0.1,0.5)\;m^{-1}$ accounts for longitudinal and lateral interaction scales of 10 m and 2 m, respectively. All predicted positions are expressed in a common coordinate frame before pairwise distances are calculated. The anticipatory adjacency matrix is constructed as
  (4) $$A_{ij}^{fut}=\{\begin{array}{c} 1,D_{ij}^{fut}\leq r_{fut},\\ 0,D_{ij}^{fut}>r_{fut}, \end{array}$$
  where $r_{fut}=1.0$ is a dimensionless interaction threshold. These settings define an interaction neighborhood rather than a collision criterion. Because $D_{ij}^{fut}=D_{ji}^{fut}$, the resulting $A^{fut}$ is a binary undirected graph. Self-loops are retained by setting $A_{ii}^{fut}$ = 1. The encoded historical representation and the selected Top-1 coarse-future representation are subsequently projected into a common latent space and concatenated along the temporal dimension, providing an extended temporal context for the SLN.
3. SLN: Micro-Kinematic Refinement Layer. The concatenated latent sequence is first re-encoded through a secondary Mamba encoder for temporal refinement. Guided by $A^{fut}$, the anticipatory graph-attention module then aggregates information from neighboring vehicles predicted to exhibit stronger future interactions with the target vehicle. Rather than independently regressing another absolute future trajectory, the residual predictor estimates a spatial displacement correction $\Delta {\hat{Y}}_{i}$. Since only the Top-1 coarse hypothesis is propagated by the ATL, the final trajectory is

(5) $${\hat{Y}}_{i}^{final}={\hat{Y}}_{i}^{c,(m_{i}^{b})}+\Delta {\hat{Y}}_{i}$$

**Figure 1:**
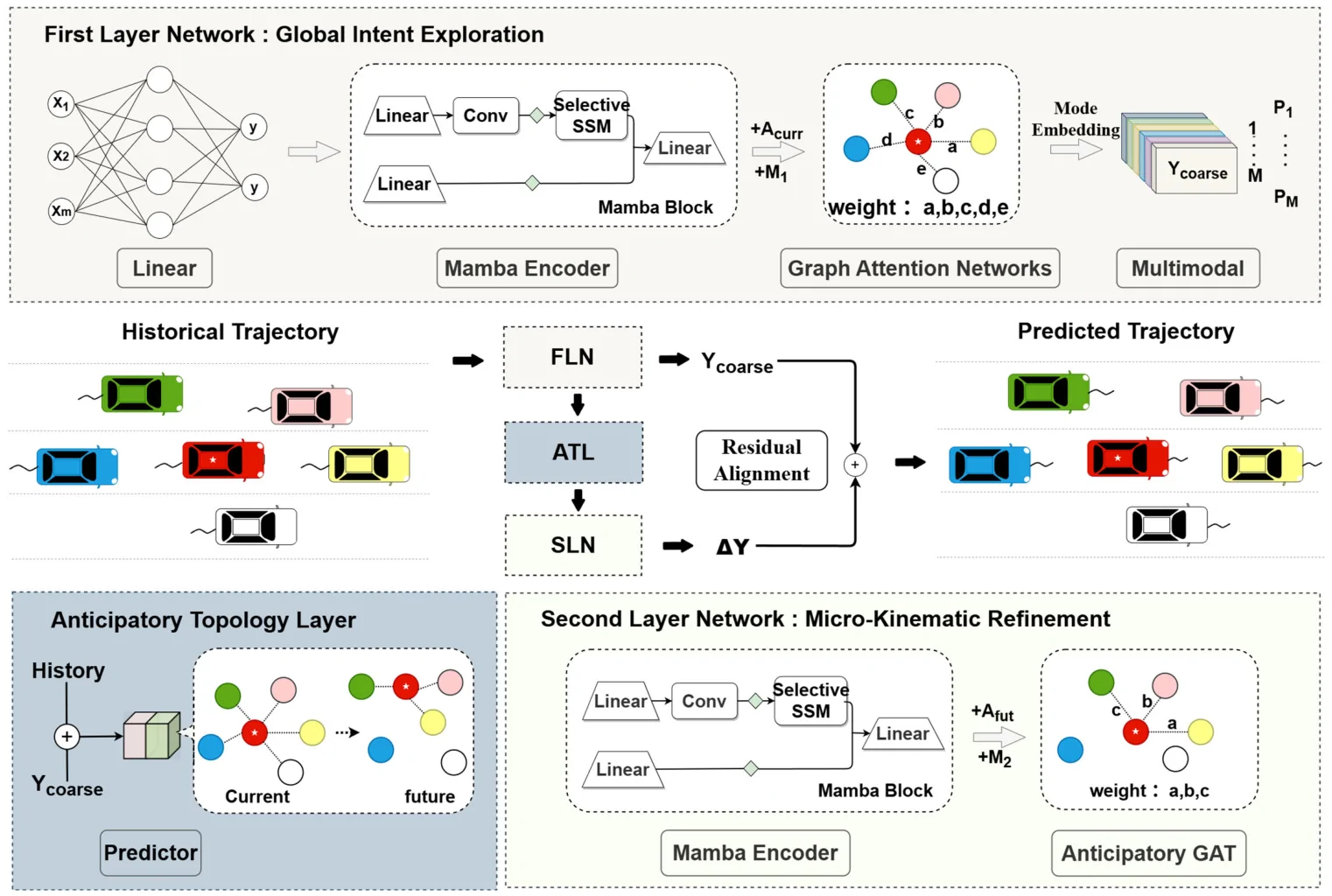
Overall architecture of the proposed HM-GAT model. The red node marked with an asterisk represents the target vehicle, while the other node colors distinguish neighboring vehicles. Labels a–e denote illustrative attention weights on the corresponding interaction edges.

Therefore, HM-GAT performs multimodal coarse candidate generation followed by Top-1 coarse-to-fine refinement. The overall architecture is illustrated in Figure 1.

### 3.3. Mamba-Based Transient Feature Encoding

**Figure 2:**
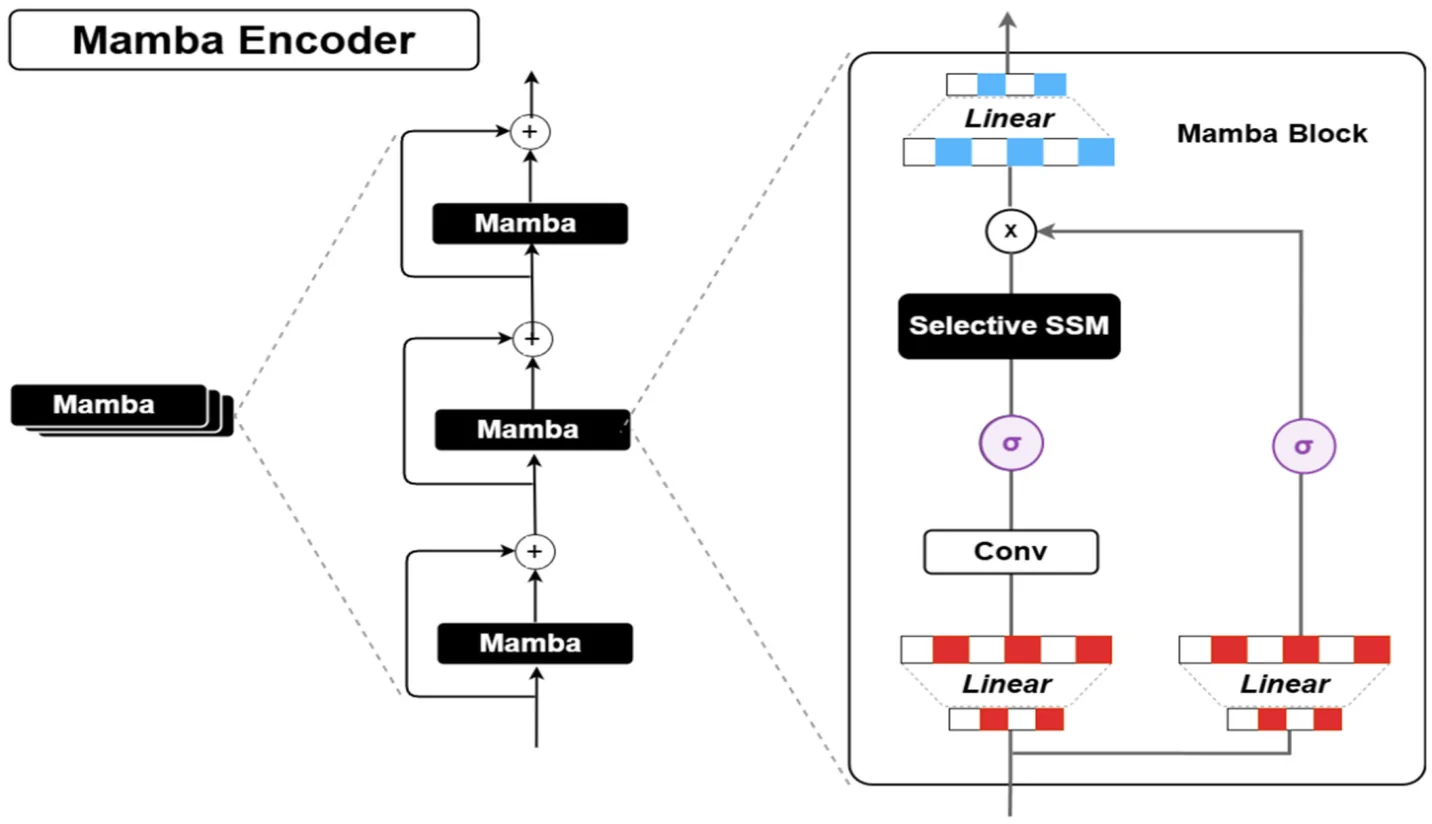
Architecture of the Mamba encoder.

In this study, a selective state space encoder is introduced into both the FLN and SLN as the core module for temporal dynamic feature extraction. Its architecture is illustrated in Figure 2. Specifically, the Mamba encoder is constructed by serially stacking three Mamba Blocks, each of which incorporates a Residual Connection structure. For each vehicle, the temporal sequence is processed chronologically using a forward causal selective scan. In the FLN, the scan proceeds from the earliest historical observation to the current observation so that the representation at the final observation step summarizes all available historical information without accessing any ground-truth future state. In the SLN, the concatenated historical–predicted representation is processed in the same chronological direction. This design preserves temporal ordering within each vehicle sequence, while graph attention separately aggregates inter-vehicle context. Through stacked selective state-space blocks and residual connections, the encoder captures long-range temporal dynamics with computational cost that scales linearly with temporal sequence length.

#### 3.3.1. Mamba Block

As illustrated in Figure 2, a Mamba Block decomposes its input representation into two parallel processing branches.

Main temporal dynamics branch: The input feature is first expanded to a higher feature dimension through a Linear layer, and subsequently fed into a one-dimensional convolution (1D Conv) layer to extract short-range local temporal patterns before selective state-space modeling. The resulting features are then activated by the σ function before being passed to the core component of the Mamba Block, namely the Selective State Space Model (Selective SSM), where the transition from continuous-time dynamics to discrete state representations is accomplished.

Dynamic gating control branch: In parallel, the original input is processed by another Linear layer followed by the σ activation function to generate a dimension-aligned gating signal. The outputs of the two branches are then fused through element-wise multiplication using the Hadamard product, enabling adaptive feature fusion [24]. Finally, the fused features are projected back to the original feature dimension through a final Linear layer and delivered to the next layer via a Residual Connection.

#### 3.3.2. Selectivity Mechanism

The core of the Mamba Block lies in its input-dependent selectivity mechanism. Given the input representation $u_{t}$, the discretization step and state-space parameters are adaptively generated according to the current input:

(6) $$\Delta_{t}=softplus\left(W_{\Delta }u_{t}+b_{\Delta }\right)$$

(7) $$B_{t}=W_{B}u_{t},C_{t}=W_{C}u_{t},$$

where $\Delta_{t}$ denotes the input-dependent discretization step, whereas $B_{t}$ and $C_{t}$ denote input-conditioned state-space parameters. To distinguish continuous-time parameters from discretized quantities, A and $B_{t}$ denote continuous-time state-space parameters, whereas ${\bar{A}}_{t}$ and ${\bar{B}}_{t}$ denote the corresponding discretized parameters. The discretized transition matrix can be expressed as

(8) $${\bar{A}}_{t}=exp\left(\Delta_{t}A\right)$$

In the selective-scan implementation, its discretized counterpart ${\bar{B}}_{t}$ is obtained by multiplying $B_{t}$ by the input-dependent step $\Delta_{t}$, with broadcasting across the corresponding channel and state dimensions. The discrete state update is then given by

(9) $$h_{t}={\bar{A}}_{t}h_{t-1}+{\bar{B}}_{t}u_{t}$$

The corresponding output is generated from the hidden state according to the selective output projection used in the Mamba implementation. Because the discretization and selective state-space parameters are conditioned on the input representation, the encoder can learn to adaptively regulate the contribution of historical information according to the observed motion context. Abrupt motion changes may cause the learned representation to emphasize more recent observations, whereas relatively stable motion may retain information over a longer temporal context. Such behavior is learned from data and should not be interpreted as a mathematically guaranteed hard state-overwrite mechanism.

### 3.4. Heterogeneous Topology-Driven Graph Attention and Adaptive Sparse Interaction

**Figure 3:**
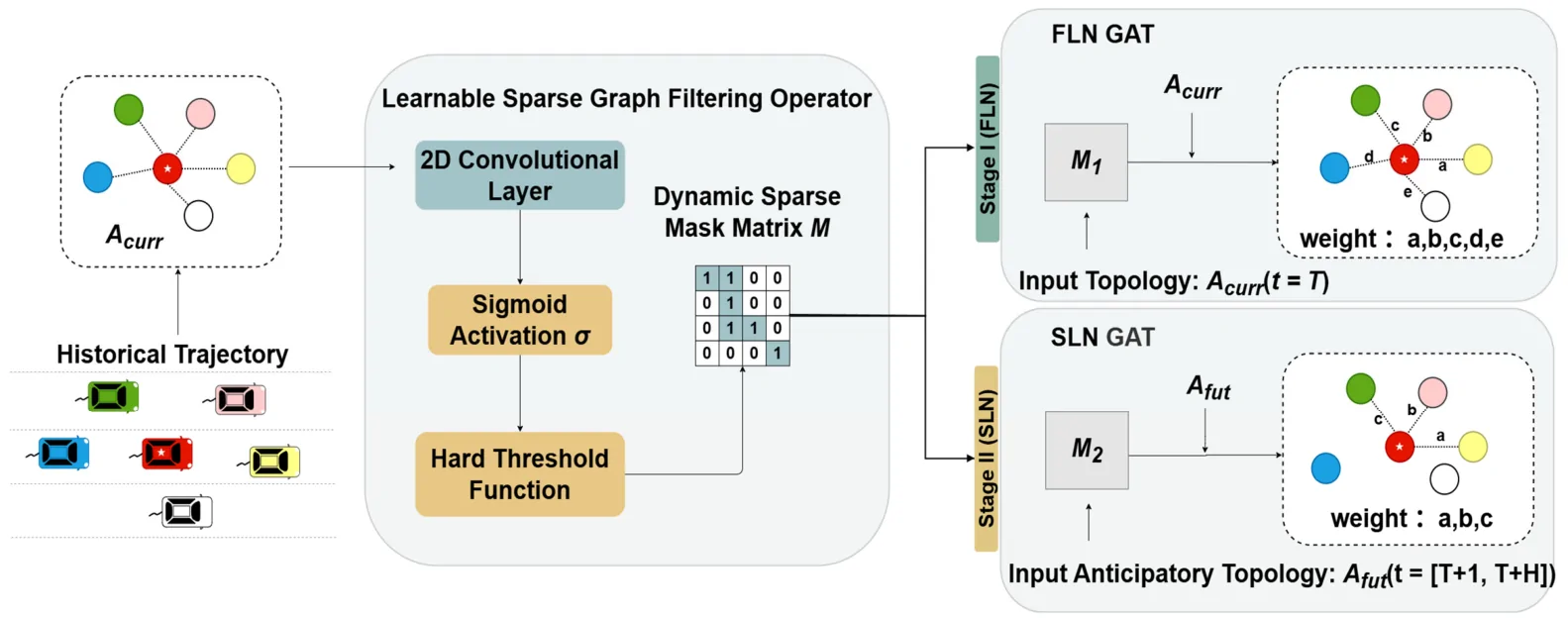
Graph attention mechanism with adaptive sparse interaction. The red node marked with an asterisk represents the target vehicle, while the other node colors distinguish neighboring vehicles. Labels a–e denote illustrative attention weights on the corresponding interaction edges.

To enhance spatial interaction modeling, HM-GAT adopts a heterogeneous dual-topology graph aggregation framework equipped with an adaptive sparsification module. As illustrated in Figure 3, sparse filtering is performed before graph-attention aggregation to suppress weakly relevant interaction edges. Within each target-centered graph, the target vehicle is placed first, followed by neighboring vehicles sorted by lateral position and then longitudinal position at the final observation time; ties are resolved using vehicle identifiers. The same ordering is retained across the historical sequence and both graph stages. This prescribed ordering standardizes the adjacency representation but does not guarantee permutation equivariance of the convolutional scoring operator. The resulting sparse masks may depend on the node ordering, and this dependence constitutes a limitation of the current implementation. Graphs are zero-padded to the maximum node count within each batch, and padded entries are excluded from convolutional scoring inputs, attention aggregation, and edge statistics using validity masks. For graph-interaction stage l∈{1,2}, where $A_{1}=A^{curr},A_{2}=A^{fut}$, a two-dimensional convolution-based scoring operator $f_{\theta_{l}}(\cdot )$ transforms the structured adjacency representation into edge-retention scores. The corresponding retention probabilities are $P_{l,ij}=\sigma \left([f_{\theta_{l}}(A_{l}){]}_{ij}\right)$. A hard binary sparse mask is then generated as

(10) $$M_{l,ij}^{hard}=\{\begin{array}{c} 1,P_{l,ij}\geq \tau ,\\ 0,P_{l,ij}<\tau , \end{array}$$

where τ tau is the sparsification threshold. In the current implementation, τ tau is a fixed hyperparameter rather than a learned parameter and is set to 0.5. Thus, the threshold itself is not optimized during training, whereas the edge-retention probabilities are learned. Since hard thresholding is non-differentiable, a Straight-Through Estimator (STE) is employed during training:

(11) $$M_{l,ij}^{STE}\;=\;\mathrm{sg}\left(M_{l,ij}^{hard}-P_{l,ij}\right)\;+\;P_{l,ij}\;$$

where sg(⋅) denotes the stop-gradient operator. During training, the straight-through mask numerically gates the unnormalized attention weights before renormalization. The forward pass therefore uses binary edge selection, while approximate gradients can propagate to the continuous retention probabilities. Inference uses the corresponding hard mask.

The sparse score should be interpreted as a learned interaction-relevance score rather than an explicit collision-risk probability. Accordingly, the sparse module does not analytically guarantee that every distant but rapidly approaching vehicle will always be retained. The possibility of prematurely removing safety-relevant edges is therefore quantitatively examined through Miss Rate, threshold-sensitivity, and risk-edge-retention experiments in Section 4.

In the FLN, under the current adjacency matrix $A^{curr}$ and sparse mask $M_{1}$, the unnormalized attention score between target vehicle i and neighboring vehicle j is computed as

(12) $${\;\;e}_{ij}^{\left(1\right)}=LeakyReLU\left[a_{1}^{⊤}\left(W_{1}h_{i}\;\|\;W_{1}h_{j}\right)\right]$$

where $h_{i}$ denotes the temporal representation extracted by the FLN Mamba encoder, and $W_{1}$ and $a_{1}$ are learnable parameters. The equivalent hard forward-pass masking and attention normalization are expressed as follows:

(13) $$e_{ij}^{\left(l\right)}=\{\begin{array}{c} e_{ij}^{\left(l\right)},A_{ij}^{\left(l\right)}M_{l,ij}^{hard}=1,\\ -\infty ,A_{ij}^{\left(l\right)}M_{l,ij}^{hard}=0, \end{array}\;\;\;\;\alpha_{ij}^{\left(l\right)}=\frac{exp(e_{ij}^{\left(l\right)})}{\sum_{k}exp(e_{ik}^{\left(l\right)})}$$

Thus, an edge removed by the sparse mask does not participate in the Softmax denominator, but rather, it merely receives a lower additive attention bias. The FLN-GAT consequently produces attention-weighted interaction representations under the observed current topology, which are used for coarse multimodal trajectory generation. After the historical and Top-1 coarse-future representations are concatenated and re-encoded by the secondary Mamba encoder, the SLN performs graph attention under $A^{fut}$ and sparse mask $M_{2}$. Unlike the FLN-GAT, whose receptive field is determined by the currently observed topology, the SLN-GAT operates on interaction relationships predicted to emerge during the future horizon. Its attention weights therefore represent the relative importance of neighboring vehicles under the anticipatory topology rather than explicit collision probabilities. The overall graph-attention mechanism with adaptive sparse interaction is illustrated in Figure 3.

### 3.5. Optimization Objective and Multi-Task Loss Formulation

To enable end-to-end joint optimization within the coarse-to-fine architecture, HM-GAT employs a multi-task objective consisting of mode classification, coarse trajectory regression, and final refinement losses. During training, the ground-truth-matched coarse mode is determined according to its average displacement error over the complete prediction horizon:

(14) $$m_{i}^{*}=\underset{m\in \{1,\ldots ,M\}}{\arg\min}\;\frac{1}{T_{f}}\sum_{\tau =1}^{T_{f}}{\left\|{\hat{y}}_{i,\tau }^{c,(m)}-y_{i,\tau }^{GT}\right\|}_{2}$$

The mode $m_{i}^{*}$ is an oracle supervision label used only during training and is distinct from the Top-1 prediction $m_{i}^{b}=\arg\underset{m}{\max}\;p_{i}^{\left(m\right)}$ used by the ATL and SLN. The mode-classification loss is

(15) $$L_{cls}=-\frac{1}{B}\sum_{i=1}^{B}\log\;p_{i}^{\left(m_{i}^{*}\right)}$$

which encourages the probability branch to assign higher probability to the ground-truth-matched candidate. The coarse trajectory regression loss is

(16) $$L_{coarse}=\frac{1}{B}\sum_{i=1}^{B}SmoothL1\left({\hat{Y}}_{i}^{c,(m_{i}^{*})},\;Y_{i}^{GT}\right)$$

Thus, $L_{cls}$ supervises the ranking of multimodal hypotheses, whereas $L_{coarse}$ supervises the geometric accuracy of the matched coarse trajectory. Importantly, the ATL and SLN do not receive the oracle mode $m_{i}^{*}$. Instead, they operate on the model-selected Top-1 trajectory ${\hat{Y}}_{i}^{c,(m_{i}^{b})}$. Therefore ${\hat{Y}}_{i}^{final}={\hat{Y}}_{i}^{c,(m_{i}^{b})}+\Delta {\hat{Y}}_{i}$ and the final refinement loss is

(17) $$L_{final}=\frac{1}{B}\sum_{i=1}^{B}SmoothL1\left({\hat{Y}}_{i}^{final},\;Y_{i}^{GT}\right)$$

This design keeps the refinement pathway consistent between training and inference and enables the SLN to learn corrections for the actual Top-1 prediction produced by the FLN.

The complete optimization objective is

(18) $$L_{\mathrm{total}}={\;\lambda }_{1}L_{\mathrm{cls}\;}+\lambda_{2}L_{\mathrm{coarse}}+\lambda_{3}L_{\mathrm{final}}$$

where the loss coefficients are fixed at $\lambda_{1}\;=\;0.3\;,\;\lambda_{2}\;=\;0.4\;,\;\lambda_{3}=0.3$. All three objectives are jointly optimized throughout training, without an epoch-dependent weighting schedule. The complete HM-GAT framework is trained end-to-end using gradient-based optimization. The current HM-GAT is primarily data-driven and does not explicitly impose an Ackermann-steering or bicycle-model constraint during optimization. Although the collision-rate evaluation does not directly establish compliance with vehicle kinematic constraints, it provides a quantitative assessment of vehicle-interaction-related risks in the predicted trajectories, helping evaluate the contribution of the proposed vehicle interaction modeling.

## 4. Experiments and Results Analysis

### 4.1. Dataset

The proposed HM-GAT model is evaluated on the NGSIM benchmark, a widely recognized large-scale real-world dataset in the fields of autonomous driving and intelligent transportation. The dataset comprises two real-world highway scenarios, namely US-101 and I-80, and provides heterogeneous traffic trajectory data containing multiple attributes, including vehicle position, velocity, acceleration, and headway distance. To construct prediction samples, continuous trajectories are segmented using a sliding-window strategy. Each sample contains an 8 s sequence, with the first 3 s used as the historical observation period and the subsequent 5 s used as the prediction horizon. The original 10 Hz trajectories are downsampled to 5 Hz, with a sliding-window stride of 1 s. Downsampling reduces redundancy between closely spaced observations and approximately halves the sequence length within the same observation–prediction window, lowering the computational and memory demands of temporal modeling. The resulting 0.2 s temporal resolution provides successive motion observations for representing changes in vehicle position and velocity, supporting the modeling of typical car-following and lane-changing dynamics while maintaining computational efficiency. To reduce potential temporal and scene-level overlap between subsets, the dataset is partitioned using a non-overlapping scene/temporal protocol rather than relying solely on target-vehicle identifiers. Continuous recording intervals assigned to the training, validation, and test sets are mutually exclusive, and a guard interval of at least 8 s is introduced at partition boundaries so that no complete observation–prediction window spans two subsets. Following preprocessing and sliding-window extraction, the training, validation, and test sets comprise 540,610, 77,230, and 154,460 samples, respectively, corresponding to 6444, 921, and 1841 distinct target vehicles. Sample counts refer to valid observation–prediction windows, while vehicle counts refer to distinct target vehicles contributing at least one valid window within each subset.

To further evaluate the generalization of HM-GAT beyond structured highway traffic, experiments are additionally conducted on the Argoverse 2 Motion Forecasting Dataset (AV2). AV2 contains highly interactive urban driving scenarios with substantially more diverse road geometries and agent behaviors than the NGSIM highway benchmark. Following the standard AV2 forecasting protocol, 5 s of historical observations are used to predict the subsequent 6 s trajectory at 10 Hz. Because the original HM-GAT is designed primarily from trajectory-derived kinematic and interaction information, no additional HD-map encoder is introduced specifically for the AV2 experiments.

### 4.2. Evaluation Metrics

Following commonly adopted evaluation protocols in multimodal motion forecasting, we employ minADE(K), minFDE(K), b−minFDE(K) and MR(K) to evaluate prediction accuracy, multimodal coverage, and confidence quality. minADE(K) measures the minimum average displacement error between the ground-truth trajectory and the K predicted trajectories over the complete prediction horizon, while minFDE(K) measures the minimum displacement error at the final prediction step. b−minFDE(K) further incorporates the confidence assigned to the selected trajectory and therefore jointly reflects trajectory accuracy and probability calibration. MR(K) denotes the proportion of samples for which none of the K predicted trajectories successfully covers the ground-truth future according to the benchmark miss criterion. For AV2, a prediction is regarded as a miss when the minimum final displacement error among the K candidates exceeds 2 m. For the NGSIM diagnostics, MR is the percentage of evaluated targets whose selected trajectory has a final displacement error greater than 2 m.

We report results for K=1 and K=6. For K=1, HM-GAT is evaluated using the highest-probability trajectory after SLN refinement, corresponding to the actual trajectory selected during inference. For K=6, the six probabilistic coarse hypotheses generated by the FLN are evaluated to quantify multimodal candidate coverage and uncertainty estimation. When more than K trajectories are available for a baseline model, the K trajectories with the highest predicted probabilities are retained following the corresponding benchmark protocol.

### 4.3. Experimental Settings

All experiments are conducted on Ubuntu 22.04 LTS using an NVIDIA GeForce RTX 4090 GPU. HM-GAT is implemented in PyTorch 2.5.1, with key hyperparameters summarized in Table 1. Training uses a random seed of 42, a fixed learning rate of 0.001, and zero weight decay. The global L2 gradient norm is clipped to a maximum of 1.0, and early stopping is not employed. For evaluation, we select the checkpoint achieving the lowest validation ADE for the final SLN Top-1 predictions.

### 4.4. Comparative Experiments

#### 4.4.1. Comparison on NGSIM

To comprehensively and objectively evaluate the trajectory prediction performance of the proposed HM-GAT model, we compare it with 11 representative state-of-the-art methods developed on the NGSIM dataset. A brief description of each baseline method is provided as follows:

1. Constant Velocity (CV): A physics-based baseline that predicts future trajectories under the assumption of constant vehicle velocity.
2. LSTM with Convolutional Social Pooling (CS-LSTM) [21]: This model is the first to introduce a convolutional social pooling mechanism into the trajectory prediction.
3. Enhanced Graph-Based Interaction-Aware Trajectory Prediction (GRIP++) [29]: This model combines fixed and dynamic graph connections to capture vehicle interactions.
4. Planning-Informed Trajectory Prediction (PiP) [35]: Proposes a prediction paradigm that tightly couples intention inference with motion planning.
5. Temporal Convolutional Networks with LSTM (TCN-LSTM) [18]: This model employs a hybrid neural network architecture that combines dilated causal convolutions with a two-layer LSTM.
6. Graph-Based Spatial-Temporal Convolutional Network (GSTCN) [30]: This model combines GCNs and CNNs to extract heterogeneous spatiotemporal features and uses a GRU-based encoder–decoder for multimodal trajectory prediction.
7. Spatiotemporal Attention LSTM (STA-LSTM) [31]: This baseline method incorporates a bidirectional spatiotemporal attention mechanism into the conventional LSTM encoder–decoder framework.
8. Dynamic Drivable Region Adaptive Multimodal Vehicle Trajectory Prediction (DACR-AMTP) [32]: This model combines multi-head self-attention with collision-risk-aware dynamic drivable region estimation to adaptively generate physically feasible multimodal trajectories.
9. Fusion of Historical Interactive Features (FHIF) [33]: This model employs multimodal factorized bilinear pooling to effectively fuse historical interaction features extracted by a multi-head attention mechanism.
10. Human-Like Trajectory Prediction (HLTP) [36]: Inspired by cognitive science, this model simulates human visual attention shifts within an LSTM decoder to improve trajectory prediction.
11. Sparse Spatial-Temporal Transformer (SSTT) [34]: Built upon the Transformer architecture, this model introduces a learnable sparse spatial interaction module together with local-global temporal modeling for trajectory prediction.

#### 4.4.2. NGSIM Comparative Results

Table 2 presents the performance comparison between the proposed HM-GAT model and multiple representative trajectory prediction methods on the NGSIM dataset. The best and second-best results are highlighted in bold and underlined, respectively. As shown in Table 2, HM-GAT achieves the lowest RMSE from the 2 s to 5 s prediction horizons as well as the lowest reported Average RMSE, ADE, and FDE among the compared methods. In particular, for the more challenging long-term prediction tasks with prediction horizons of 3 s, 4 s, and 5 s, the RMSE is reduced by 28.9%, 33.9%, and 41.5%, respectively, compared with the best-performing baseline model. Furthermore, compared with the recently proposed SSTT (2025), HM-GAT achieves reductions of 15.8% and 32.1% in ADE and FDE, respectively. These results indicate that HM-GAT is more effective at modeling complex spatiotemporal interactions, thereby achieving more accurate long-term trajectory prediction.

**Figure 4:**
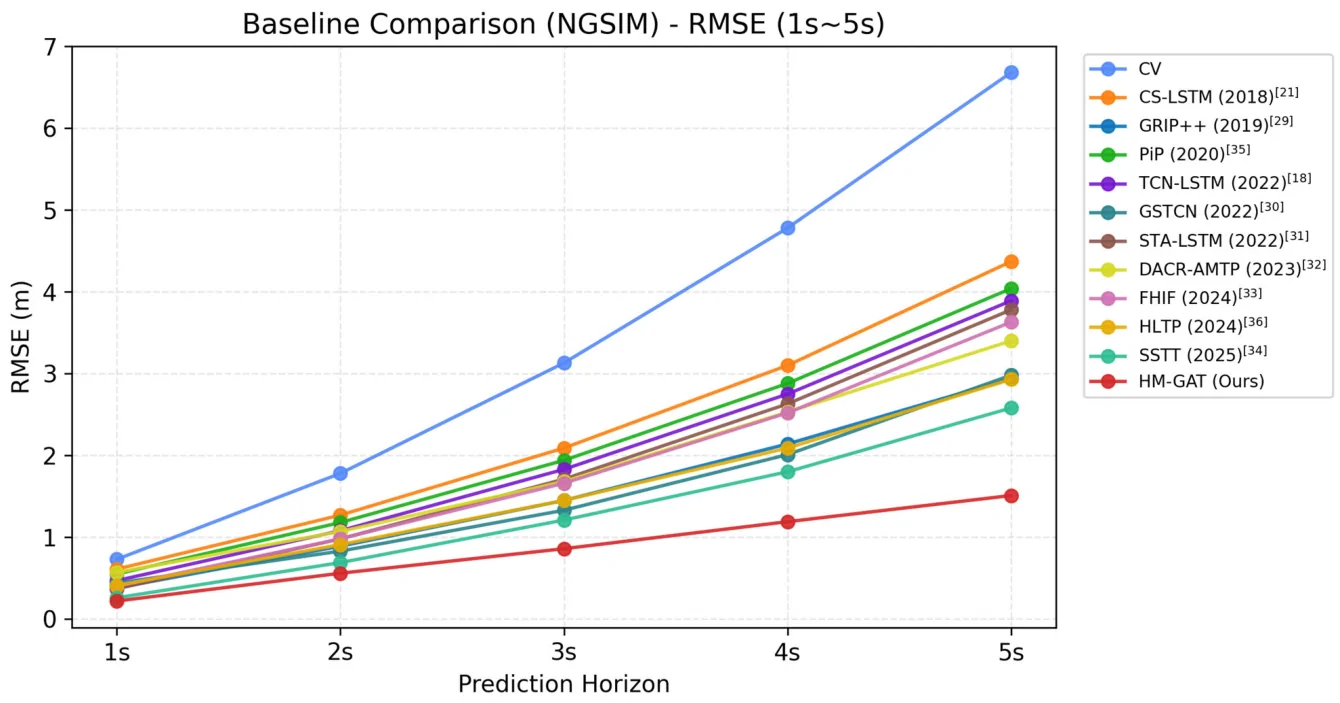
RMSE comparison of different models under different prediction horizons [18,21,29,30,31,32,33,34,35,36].

Figure 4 visualizes the horizon-specific RMSE values in Table 2. The gap between HM-GAT and the compared baselines becomes more pronounced toward the end of the prediction horizon, although HM-GAT is slightly behind SSTT at 1 s. These curves describe error growth with forecast horizon.

#### 4.4.3. Comparison on Argoverse 2

To evaluate whether the proposed model generalizes to highly interactive urban traffic, HM-GAT is further compared with modern motion forecasting architectures on the AV2 vehicle subset. QCNet [37] is included as a representative attention-based trajectory predictor, while Trajectory Mamba [26] is included as a directly related selective state-space forecasting method. FINet [27] is also included as a representative future-aware interaction modeling baseline for motion forecasting. Table 3 evaluates the final Top-1 trajectory after SLN refinement, whereas Table 4 evaluates the six probabilistic coarse hypotheses generated by the FLN. For the present survey comparison, Table 3 also reports measured inference latency, complementing the prediction metrics with an assessment of computational efficiency.

Table 3 and Table 4 show that HM-GAT achieves competitive prediction performance without using HD-map priors. Its Top-1 accuracy is close to QCNet and Trajectory Mamba, while its minFDE(6) and b-minFDE(6) rank second among the compared methods. HM-GAT also achieves the lowest measured inference latency in Table 3, reducing latency by 65.5%, 41.2%, and 16.7% relative to QCNet, Trajectory Mamba, and FINet, respectively. Although FINet provides better Top-1 accuracy, HM-GAT offers a favorable accuracy–latency trade-off with reduced dependence on map information under the evaluated configuration.

### 4.5. Ablation Study

#### 4.5.1. Ablation Settings

To systematically investigate the contributions of the major architectural components and the hierarchical coarse-to-fine prediction mechanism of HM-GAT, we conduct both component-level ablation experiments and stage-wise prediction analyses. The component-level experiments examine the effects of temporal modeling, graph-based interaction modeling, adaptive sparsification, anticipatory future topology, and second-stage refinement.

1. Case I (*w*/*o* MLP replacing FLN Mamba): The Mamba encoder in the FLN is replaced with a multilayer perceptron (MLP).
2. Case II (*w*/*o* FLN GAT): The graph attention module in the FLN is removed, eliminating spatial interaction modeling in the first stage.
3. Case III (*w*/*o* FLN linear candidate): The learned FLN candidate generator is replaced with linear extrapolation before the remaining prediction pipeline.
4. Case IV (*w*/*o* MLP replacing SLN Mamba): The Mamba encoder in the SLN is replaced with a multilayer perceptron (MLP).
5. Case V (*w*/*o* SLN GAT): The GAT module in the SLN is removed, thereby disabling second-stage interaction aggregation guided by the anticipatory future topology.
6. Case VI (*w*/*o* Anticipatory Future Topology): The anticipatory future topology is replaced with the current topology, while the remaining architecture is unchanged.
7. Case VII (*w*/*o* SLN, FLN-only): The entire SLN is removed, reducing HM-GAT to a single-stage trajectory prediction framework.
8. Case VIII (*w*/*o* Sparse Filtering): Disable the adaptive sparse interaction mask in the two graph attention stages, keeping the rest of the structure unchanged.

#### 4.5.2. Ablation Results

Table 5 compares eight ablation variants with the complete HM-GAT, which achieves the lowest errors across all reported metrics. Relative to the full model, replacing the Mamba encoder with an MLP in the FLN and SLN increases ADE by 47.5% and 41.3%, respectively, supporting the contribution of temporal encoding in both stages. Replacing the FLN with linear extrapolation increases ADE by 82.5%, highlighting the importance of learned coarse prediction. Removing the entire SLN increases FDE by 112.2%, indicating the substantial contribution of second-stage refinement to endpoint accuracy. Removing the FLN GAT increases FDE by 16.9%, demonstrating the value of current-topology interaction modeling. Removing the SLN GAT increases FDE by 46.6%, supporting the combined contribution of second-stage interaction aggregation and anticipatory-topology guidance. To further isolate the effect of topology choice, Case VI retains the SLN GAT but replaces the anticipatory future topology with the current topology, with the remaining architecture unchanged. This substitution increases ADE and FDE by 53.8% and 41.2%, respectively. The controlled comparison therefore provides direct empirical evidence for the benefit of anticipatory topology within the same graph-attention refinement architecture. Disabling sparse filtering increases ADE and FDE by 21.3% and 11.5%, respectively, suggesting that selective interaction aggregation improves prediction accuracy. Together, these results support the complementary contributions of temporal encoding, graph-based interaction modeling, and hierarchical refinement within the evaluated framework.

**Figure 5:**
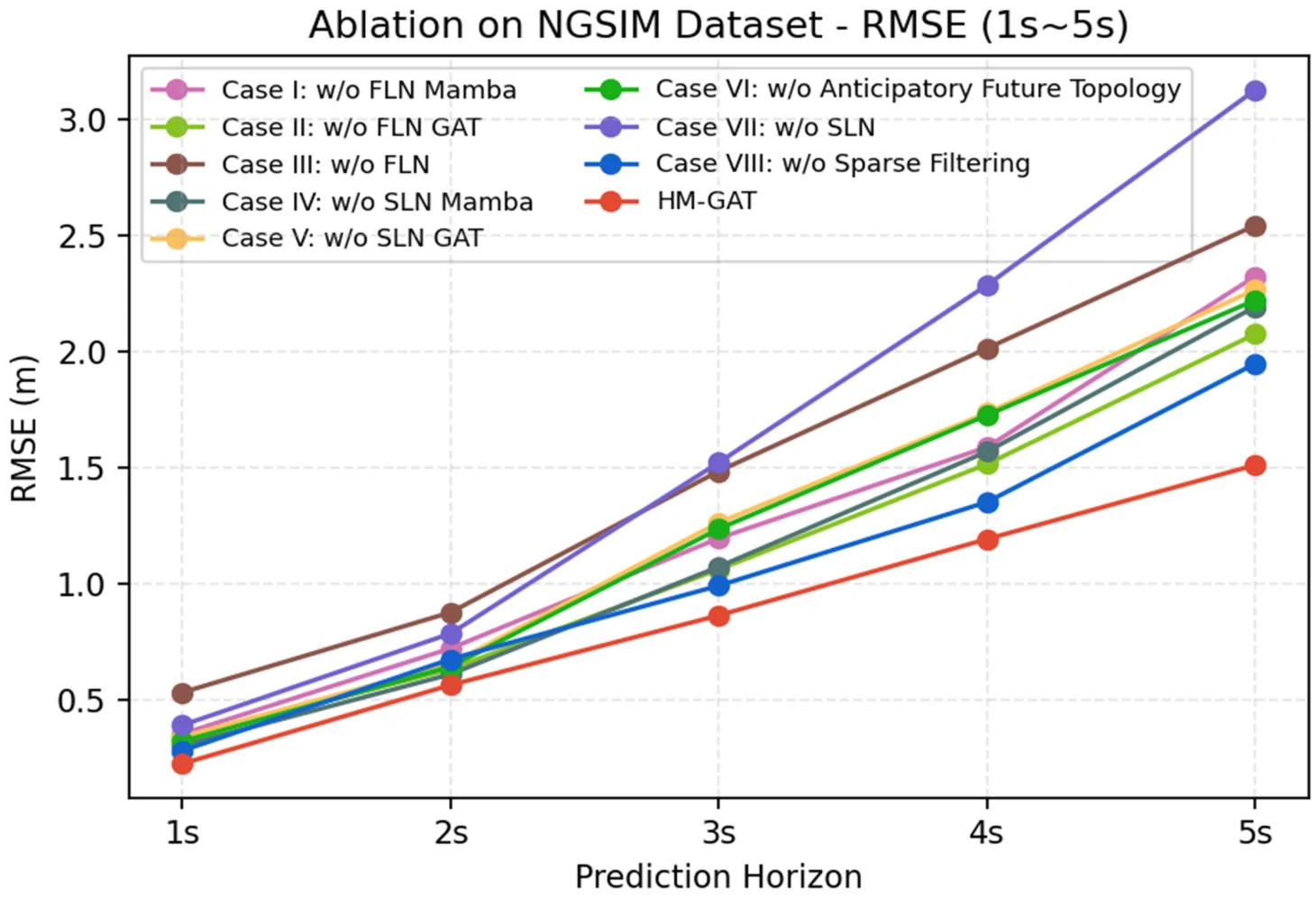
RMSE curves of the ablation variants over different prediction horizons.

**Figure 6:**
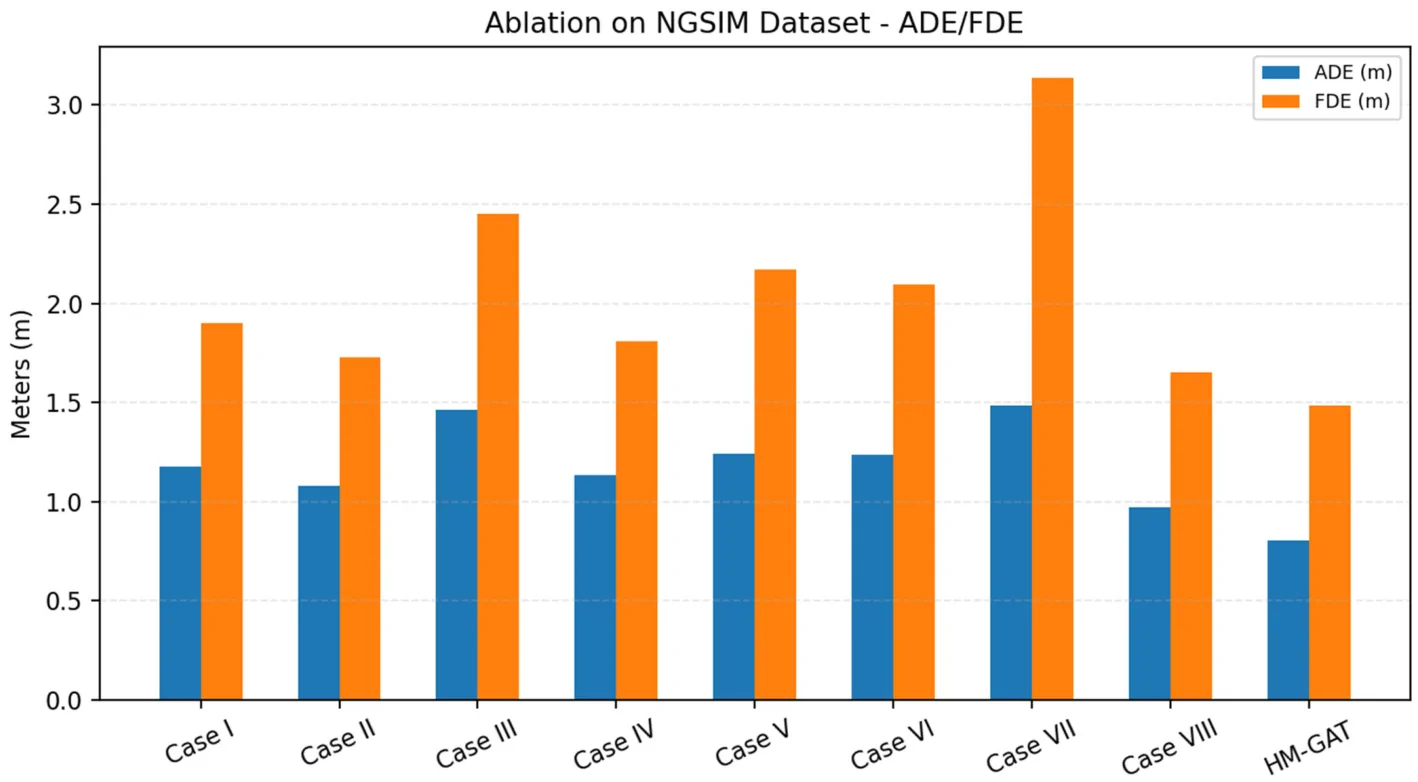
Comparison of ADE and FDE among the ablation variants.

The RMSE curves of the ablation variants over prediction horizons from 1 s to 5 s shown in Figure 5, together with the ADE/FDE performance comparison presented in Figure 6, further demonstrate that HM-GAT consistently outperforms all ablation variants across all evaluation metrics at every prediction horizon. These results further highlight the contribution of each core spatiotemporal component to the overall performance of the proposed HM-GAT framework.

#### 4.5.3. Stage-Wise Analysis of the Hierarchical FLN–SLN Framework

To further evaluate the hierarchical refinement mechanism, we retrain the complete HM-GAT model and conduct a dedicated stage-wise analysis using the resulting checkpoint. For each target vehicle, the highest-probability hypothesis among the six FLN candidates is selected and subsequently refined through anticipatory-topology-guided interaction modeling and the SLN. The intermediate FLN Top-1 trajectory and the corresponding final prediction are evaluated on identical test samples, allowing a direct assessment of refinement within the same trained model.

Table 6 shows that second-stage refinement reduces prediction errors across all evaluated horizons. Compared with the FLN Top-1 output, the final prediction reduces ADE and FDE by 45.5% and 50.7%, respectively, while the 5 s RMSE decreases by 50.3%. MR also falls from 87.26% to 6.44%. These paired results demonstrate the contribution of the hierarchical refinement stage to improving both overall trajectory accuracy and endpoint reliability. They support the effectiveness of combining coarse prediction with anticipatory-topology-guided interaction aggregation and residual correction.

### 4.6. Sensitivity Analysis of the Sparse Interaction Filter

To examine the trade-off between interaction sparsity and prediction accuracy, we vary the inference threshold τ from 0.1 to 0.9 while keeping the trained checkpoint and test samples fixed and include a filtering-disabled reference. Table 7 reports FDE, Top-1 MR, retained-edge ratios in the current and future graphs, risk-pair retention, and full-pipeline latency. Current- and future-edge ratios are normalized by their respective unfiltered candidate graphs. Risk-pair retention is measured against all reference risk pairs identified using an independent close-approach criterion, thereby accounting for exclusions during both candidate selection and filtering. Ground-truth futures are used only for offline risk labeling.

Among the tested settings, τ = 0.5 achieves the lowest FDE and MR, reducing them by 10.3% and 47.5%, respectively, compared with disabling filtering. At this threshold, current- and future-graph edge counts decrease by 55.08% and 44.82%, while risk-pair retention decreases by only 0.35 percentage points. This suggests that moderate filtering removes many edges while preserving most of the reference risk pairs represented in the initial graphs. More aggressive filtering introduces a clear trade-off: at τ = 0.9, risk-pair retention falls to 51.26%, accompanied by higher FDE and MR. Thus, fewer retained edges do not necessarily yield better predictions. Latency remains at 3.40 ms across the tested thresholds, slightly above the 3.30 ms filtering-disabled reference, indicating that the observed benefit concerns prediction accuracy and selective interaction retention rather than a measured inference speedup.

### 4.7. Trajectory Prediction Visualization

To qualitatively evaluate the prediction performance of different models in highly dynamic and complex vehicular environments, we visualize the multimodal intention distributions generated by HM-GAT, together with the predicted trajectories of HM-GAT and the baseline methods under diverse traffic scenarios. Four representative driving scenarios are considered, including normal driving, leading vehicle deceleration, lane changing, and traffic congestion.

#### 4.7.1. Visualization of Multimodal Intention Distribution

**Figure 7:**
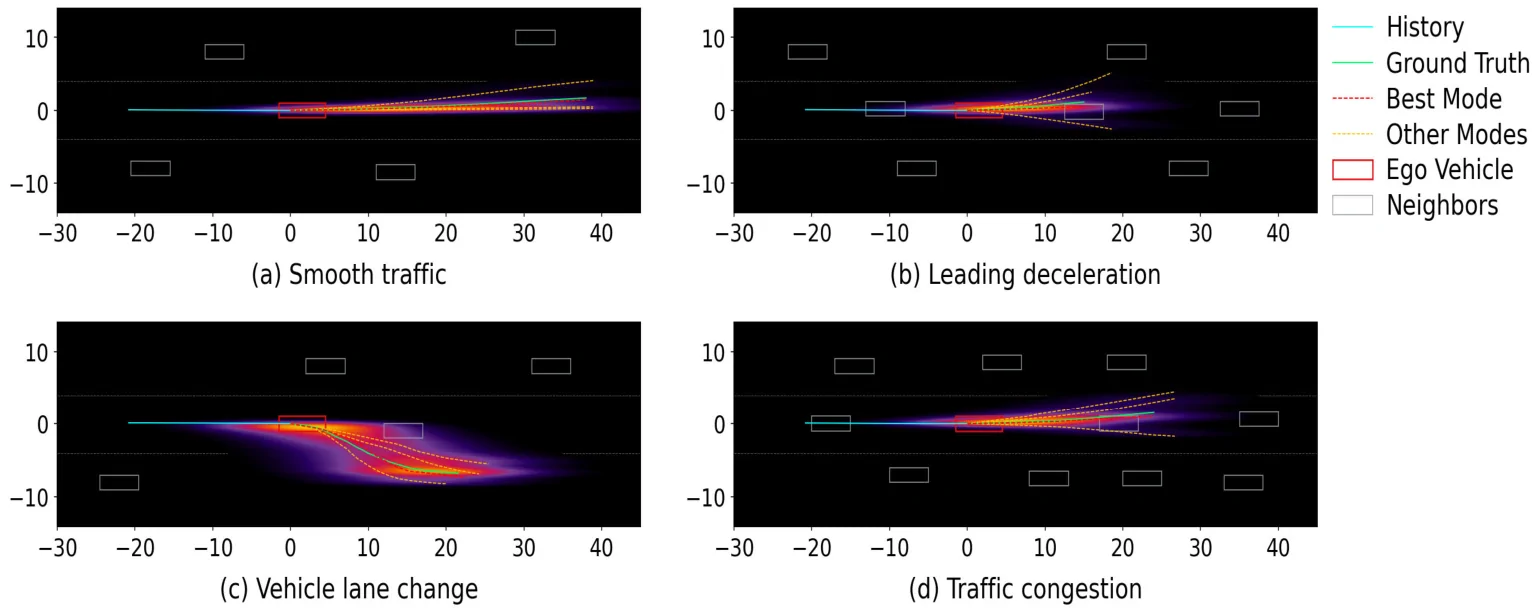
Visualization of multimodal intention distributions: (**a**) smooth traffic; (**b**) leading-vehicle deceleration; (**c**) vehicle lane change; (**d**) traffic congestion.

In Figure 7, blue and green solid lines denote historical and ground-truth future trajectories, respectively, while red and yellow dashed lines represent the selected prediction and alternative hypotheses. The probability heatmap is concentrated along the driving direction during normal driving (Figure 7a) but shows greater lateral dispersion and multiple plausible branches during lane changes (Figure 7c). Shorter longitudinal travel distances in the deceleration (Figure 7b) and congestion (Figure 7d) scenarios reflect reduced vehicle motion. Across these examples, the selected predictions lie within high-probability regions and closely follow the ground truth, illustrating HM-GAT’s ability to capture diverse motion patterns and vehicle interactions.

#### 4.7.2. Visualization Comparison of Predicted Trajectories in Complex Interaction Scenarios

**Figure 8:**
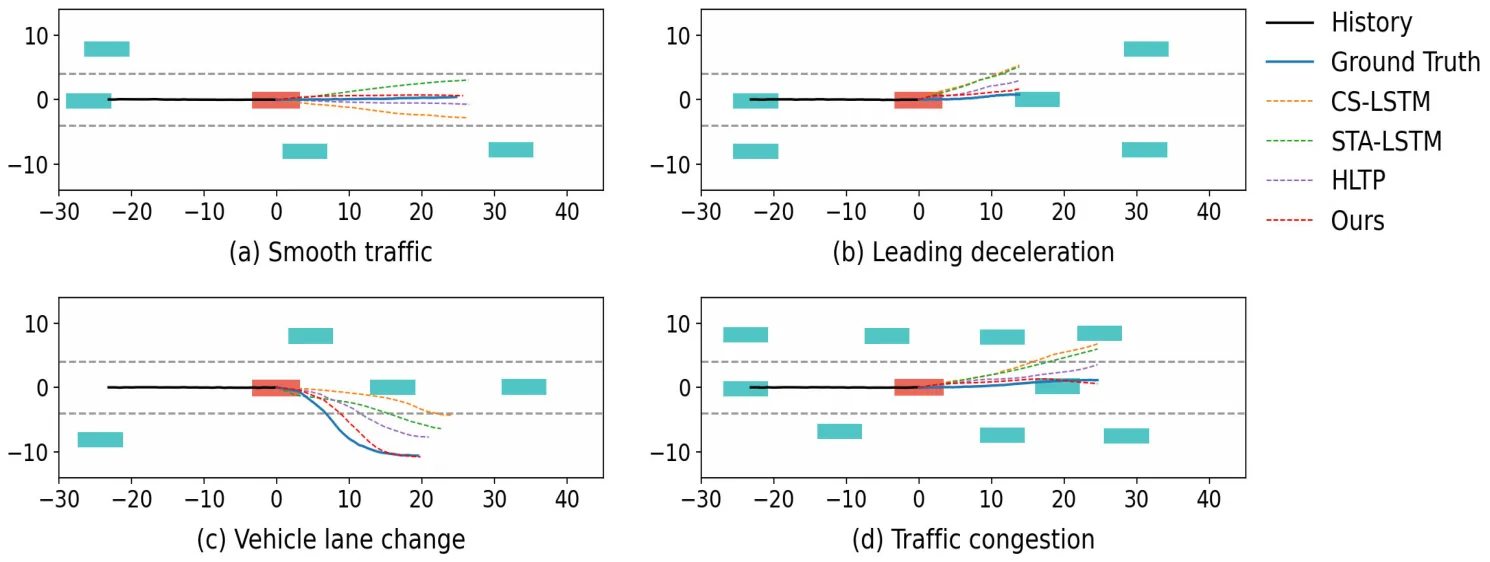
Visualization comparison of predicted trajectories of different models in complex interaction scenarios: (**a**) smooth traffic; (**b**) leading-vehicle deceleration; (**c**) vehicle lane change; (**d**) traffic congestion.

In Figure 8, the black solid line represents the historical trajectory, the blue solid line denotes the ground-truth future trajectory, and the dashed lines of different colors correspond to the predicted trajectories generated by different models, with the red dashed line representing the proposed HM-GAT model. Overall, in the normal driving scenario shown in Figure 8a, the predicted trajectories of most models closely follow the ground-truth trajectory, indicating that extrapolating future motion based on historical driving patterns is relatively straightforward. However, in the traffic congestion scenario (Figure 8d) and the lane-changing scenario (Figure 8c), where vehicle density is high and interaction relationships are more complex, several baseline methods exhibit noticeable lateral deviations from the ground-truth trajectory. This suggests that relying solely on single-stage temporal modeling or relatively weak interaction representations is insufficient to maintain accurate long-term trajectory prediction in highly coupled traffic environments. In the leading vehicle deceleration scenario (Figure 8b), the target vehicle exhibits a clear deceleration trend, with the spacing between successive future trajectory points gradually decreasing. Several baseline methods produce predictions that deviate prematurely from the ground-truth trajectory.

### 4.8. Effect of Interaction Modeling on Collision Rate

To investigate the contribution of interaction modeling to collision-related prediction outcomes, Table 8 compares HM-GAT with variants that remove the GAT module from the FLN, the SLN, or both stages. Removing the FLN GAT disables current-topology interaction aggregation, whereas removing the SLN GAT disables aggregation guided by the anticipatory future topology. FDE and collision rate (CR) are reported together to assess endpoint accuracy and collision-related behavior.

HM-GAT achieves the lowest FDE and CR among the evaluated variants. Compared with removing both GAT modules, the complete model reduces FDE by 37.0% and CR by 60.5%, supporting the contribution of graph-based interaction modeling. Relative to removing the FLN or SLN GAT individually, HM-GAT reduces CR by 37.5% and 31.8%, respectively, indicating that interaction aggregation in both stages contributes to lower collision rates. The two individual ablations also produce different metric rankings: removing the SLN GAT yields a higher FDE but a slightly lower CR than removing the FLN GAT. The differing FDE and CR rankings of the individual ablations suggest that endpoint accuracy and collision-related behavior capture complementary aspects of prediction quality. Together, these results support the combined interaction design and provide quantitative evidence of its contribution to reducing predicted inter-vehicle conflicts in the offline evaluation.

### 4.9. Robustness Analysis Under Real-World Vehicular Edge Scenarios

To validate the deployment reliability of the proposed HM-GAT model in complex and highly uncertain real-world vehicular environments, a series of rigorous robustness stress tests are conducted under various non-ideal and extreme operating conditions. The corresponding experimental settings are defined as follows:

1. Traffic Density: Traffic conditions are grouped by the number of valid neighboring vehicles within the target-centered region of ±20 m longitudinally and ±5 m laterally at the final observed timestamp. Excluding the target vehicle, samples with at most four neighbors form the low-density group, while those with at least eight form the high-density group.
2. Lane-Changing Scenario: Lane-changing, a highly dynamic and long-tail driving behavior characterized by strong irregularity, is extracted for independent evaluation.
3. Noise Interference: To simulate perception errors encountered in real-world environments, independent zero-mean Gaussian noise is added to valid historical position coordinates and scalar velocities, with standard deviations of 0.5 m and 0.2 m/s, respectively, after conversion to physical units and before normalization.
4. Missing Data: To simulate sensor data loss, each historical timestamp in a test sample is independently masked with probability 0.20, using the same mask across all vehicles and input features. Internal gaps are filled by linear interpolation, while boundary gaps use the nearest retained historical observation. Masks removing all observations are resampled, and only historical data are used for imputation.

**Figure 9:**
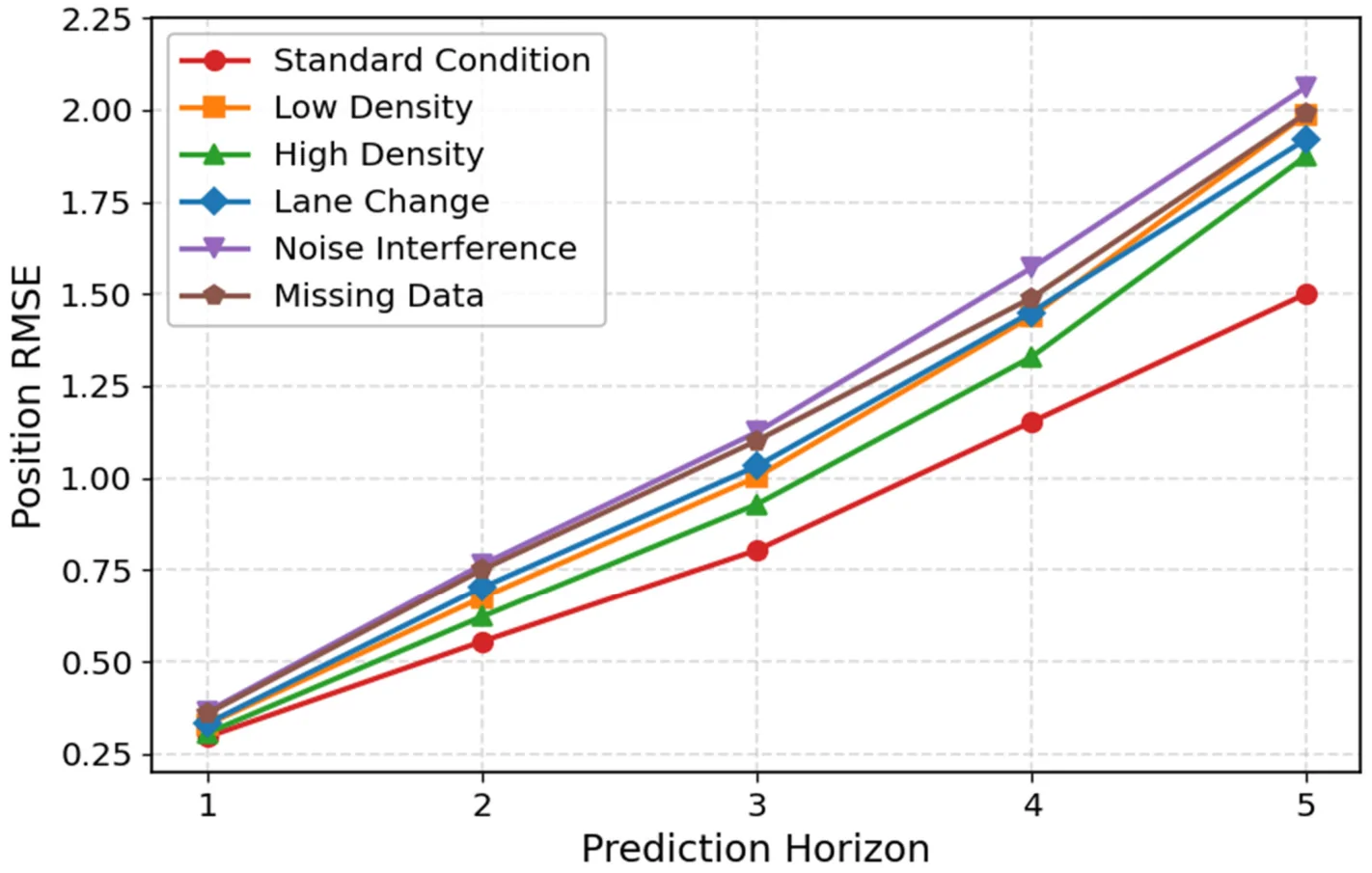
RMSE of HM-GAT over different prediction horizons under different operating conditions.

As illustrated in Figure 9, the robustness of the proposed HM-GAT model is comprehensively evaluated by taking the Standard Condition as the reference. Under different traffic density conditions, the model exhibits a higher RMSE in low-density traffic than in high-density traffic. This is because dense traffic provides richer interaction relationships and stronger spatial constraints, which facilitate more accurate trajectory inference. In the lane-changing scenario, the prediction error is relatively higher because lane changing is a highly dynamic long-tail driving behavior characterized by limited training samples and complex motion patterns. In addition, both missing data, which result in discontinuous observation information, and Gaussian noise, which degrades the quality of perception data, lead to increased prediction errors. Overall, despite complex traffic interactions and degraded perception information, the performance degradation of HM-GAT remains relatively limited, suggesting that the proposed hierarchical spatiotemporal modeling framework has some robustness and generalization capability.

### 4.10. Complexity and Hardware Efficiency

As reported in Table 9, HM-GAT achieves the lowest inference latency among the evaluated implementations while maintaining competitive prediction accuracy. Although CS-LSTM has the fewest parameters and FLOPs, its measured latency is higher, illustrating that arithmetic operation count alone does not determine inference speed. Compared with SSTT, HM-GAT reduces parameter count, FLOPs, and latency by 47.5%, 58.8%, and 26.1%, respectively, while reducing Average RMSE by 33.1%. To further examine the temporal encoder, HT-GAT replaces the Mamba modules with Transformers within the hierarchical framework. Relative to HT-GAT, HM-GAT reduces parameter count by 64.4%, FLOPs by 68.9%, and latency by 43.3%, with a modest increase in Average RMSE from 0.83 to 0.87 m. These results highlight a favorable accuracy–efficiency trade-off for the Mamba-based design under the evaluated configuration.

## 5. Conclusions

To address the key challenges of temporal memory degradation, attention dispersion, and long-term error accumulation in multi-agent trajectory prediction for highly dynamic vehicular environments, this paper proposes a hierarchical coarse-to-fine spatiotemporal optimization framework, termed HM-GAT. The proposed framework introduces the linear-complexity Mamba architecture into the temporal encoding stage, enabling efficient and accurate modeling of transient driving maneuvers, such as emergency obstacle avoidance, with low inference latency.

Furthermore, a dual-layer heterogeneous graph attention mechanism, together with an adaptive sparse filtering operator and an anticipatory future graph, is designed to alleviate attention dispersion in dense traffic scenarios while enhancing the perception of potential collision interactions. In addition, the collaborative optimization of modality classification and residual regression, combined with an end-to-end multitask learning framework, further improves long-term trajectory prediction accuracy. Experiments show favorable long-horizon accuracy on NGSIM and competitive performance on AV2.

Despite the demonstrated performance, several aspects warrant further investigation. First, the anticipatory interaction topology depends on the reliability of the coarse predictions generated by the FLN. Since only the Top-1 hypothesis is used for topology construction, safety-relevant interactions arising exclusively under lower-probability alternatives may remain unrepresented. Second, the current HM-GAT framework is data-driven and does not explicitly enforce vehicle kinematic constraints. It therefore provides no mathematical guarantee of compliance with non-holonomic constraints, and predicted trajectories may still exceed the physical actuation limits of passenger vehicles despite exhibiting low collision rates. The CR evaluation quantifies inter-vehicle conflicts and does not establish kinematic feasibility. Future work will investigate uncertainty-aware anticipatory topology construction incorporating multiple hypotheses to improve the coverage and stability of future-interaction modeling. In addition, physics-aware trajectory modeling and physically constrained refinement will be explored to enhance the kinematic feasibility of predicted trajectories, particularly in highly dynamic driving scenarios.

## Figures and Tables

**Table 1:** Experimental hyperparameter settings.

Parameter Name	NGSIM	Argoverse 2
Historical duration	3 s	5 s
Prediction duration	5 s	6 s
Sampling frequency	5 Hz	10 Hz
GAT attention heads	8	8
Number of modes M	6	6
Training epochs	100	100
learning rate	0.001	0.001
Optimizer	Adam	Adam
Sparse threshold τ	0.5	0.5
Batch size	128	128
Mamba hidden state dimension	256	256

**Table 2:** Performance comparison of RMSE, ADE, and FDE for different models on the NGSIM dataset.

Year	Model	RMSE (m)					Average RMSE (m)	ADE (m)	FDE (m)
		1 s	2 s	3 s	4 s	5 s			
	CV	0.73	1.78	3.13	4.78	6.68	3.42	-	-
2018	CS-LSTM	0.61	1.27	2.09	3.10	4.37	2.29	2.29	3.34
2019	GRIP++	0.38	0.89	1.45	2.14	2.94	1.56	1.61	-
2020	PiP	0.55	1.18	1.94	2.88	4.04	2.12	2.18	-
2022	TCN-LSTM	0.47	1.08	1.83	2.75	3.89	2.00	2.01	3.25
2022	GSTCN	0.44	0.83	1.33	2.01	2.98	1.52	1.52	-
2022	STA-LSTM	0.37	0.98	1.71	2.63	3.78	1.89	1.89	3.16
2023	DACR-AMTP	0.57	1.07	1.68	2.53	3.40	1.85	1.61	3.13
2024	FHIF	0.40	0.98	1.66	2.52	3.63	1.84	1.84	-
2024	HLTP	0.41	0.91	1.45	2.09	2.93	1.56	1.06	2.47
2025	SSTT	**0.20**	0.69	1.21	1.80	2.58	1.30	0.95	2.18
	HM-GAT	0.22	**0.56**	**0.86**	**1.19**	**1.51**	**0.87**	**0.80**	**1.48**

**Table 3:** Final Top-1 prediction performance.

Method	minADE_1_	minFDE_1_	MR_1_	Latency (ms)
QCNet	1.69	4.30	0.59	29
Trajectory Mamba	1.66	4.24	0.57	17
FINet	1.60	4.02	0.55	12
HM-GAT (SLN Final)	1.65	4.29	0.58	10

**Table 4:** Multimodal prediction quality.

Method	K	minADE_6_	minFDE_6_	MR_6_	b-minFDE_6_
QCNet	6	0.65	1.31	0.16	1.86
Trajectory Mamba	6	0.62	1.24	0.17	1.89
FINet	6	0.69	1.28	0.15	1.93
HM-GAT (FLN Final)	6	0.66	1.27	0.16	1.88

**Table 5:** Comparison of RMSE, ADE, and FDE among the ablation variants.

		Case I	Case II	Case III	Case IV	Case V	Case VI	Case VII	Case VIII	HM-GAT
RMSE (m)	1 s	0.35	0.34	0.53	0.30	0.34	0.32	0.39	0.28	0.22
	2 s	0.72	0.63	0.88	0.61	0.65	0.64	0.79	0.67	0.56
	3 s	1.20	1.06	1.48	1.07	1.26	1.23	1.52	0.99	0.86
	4 s	1.59	1.51	2.01	1.56	1.73	1.72	2.28	1.35	1.19
	5 s	2.32	2.07	2.54	2.19	2.27	2.21	3.17	1.94	1.51
ADE (m)		1.18	1.08	1.46	1.13	1.24	1.23	1.49	0.97	0.80
FDE (m)		1.90	1.73	2.45	1.81	2.17	2.09	3.14	1.65	1.48

**Table 6:** Stage-wise comparison of FLN Top-1 and SLN-final predictions.

Prediction Output	RMSE-1 s	RMSE-2 s	RMSE-3 s	RMSE-4 s	RMSE-5 s	ADE	FDE	MR (%)
FLN Top-1	0.37	0.76	1.49	2.14	3.08	1.45	3.02	87.26
SLN final prediction	0.22	0.58	0.84	1.22	1.53	0.79	1.49	6.44

**Table 7:** Effect of the inference-time sparse-filter threshold on prediction accuracy, interaction retention, and latency.

Threshold Setting	FDE (m)	MR (%)	Current Edges (%)	Future Edges (%)	Risk Retained (%)	Latency (ms)
Filtering disabled	1.65	10.36	100.00	100.00	90.00	3.30
tau = 0.1	1.54	8.04	95.82	96.60	90.00	3.40
tau = 0.3	1.51	6.69	72.13	77.36	89.99	3.40
tau = 0.5	1.48	5.44	44.92	55.18	89.65	3.40
tau = 0.7	1.49	7.30	25.54	38.42	84.81	3.40
tau = 0.9	1.75	28.27	11.69	20.17	51.26	3.40

**Table 8:** Effect of graph interaction modeling on endpoint accuracy and collision rate. FDE is measured in meters.

Model	Interaction Mechanism	FDE	CR (%)
*w*/*o* FLN GAT	No FLN GAT	1.73	2.40
*w*/*o* SLN GAT	No SLN GAT	2.17	2.20
*w*/*o* GAT	No FLN and SLN GAT	2.35	3.80
HM-GAT	Full Model	1.48	1.50

**Table 9:** Comparison of computational efficiency and inference latency among different models.

Model	Core Architecture	Parameter (M)	FLOPs (G)	Inference Latency (ms)	Average RMSE (m)
CS-LSTM	LSTM + Convolutional Social Pooling	0.4	0.3	8.5	2.29
HLTP	Vision-Aware Encoding + GRU Decoder	2.4	1.9	7.2	1.56
SSTT	Sparse Spatio-Temporal Transformer	4.0	3.4	4.6	1.30
HT-GAT	Transformer + Hierarchical Sparse GAT	5.9	4.5	6.0	0.83
HM-GAT	Mamba SSM + Hierarchical Sparse GAT	2.1	1.4	3.4	0.87

## Data Availability

The public benchmark datasets used in this study are available from their respective original sources. Additional information supporting the reported results may be made available from the corresponding author upon reasonable request.
